# The Role of Microbial Amino Acid Metabolism in Host Metabolism

**DOI:** 10.3390/nu7042930

**Published:** 2015-04-16

**Authors:** Evelien P. J. G. Neis, Cornelis H. C. Dejong, Sander S. Rensen

**Affiliations:** Department of Surgery, Maastricht University Medical Center, PO Box 616, 6200 MD Maastricht, The Netherlands; E-Mails: chc.dejong@mumc.nl (C.H.C.D.); s.rensen@maastrichtuniversity.nl (S.S.R.)

**Keywords:** amino acids, short-chain fatty acids, gut microbiota, obesity, type 2 diabetes mellitus

## Abstract

Disruptions in gut microbiota composition and function are increasingly implicated in the pathogenesis of obesity, insulin resistance, and type 2 diabetes mellitus. The functional output of the gut microbiota, including short-chain fatty acids and amino acids, are thought to be important modulators underlying the development of these disorders. Gut bacteria can alter the bioavailability of amino acids by utilization of several amino acids originating from both alimentary and endogenous proteins. In turn, gut bacteria also provide amino acids to the host. This could have significant implications in the context of insulin resistance and type 2 diabetes mellitus, conditions associated with elevated systemic concentrations of certain amino acids, in particular the aromatic and branched-chain amino acids. Moreover, several amino acids released by gut bacteria can serve as precursors for the synthesis of short-chain fatty acids, which also play a role in the development of obesity. In this review, we aim to compile the available evidence on the contribution of microbial amino acids to host amino acid homeostasis, and to assess the role of the gut microbiota as a determinant of amino acid and short-chain fatty acid perturbations in human obesity and type 2 diabetes mellitus.

## 1. Introduction

Gut microbiota and its metabolites have a pivotal role in the maintenance of physiologic and metabolic homeostasis of the host [1]. The host and its indigenous microbiota exhibit numerous mutually beneficial and cooperative interactions. In particular, the metabolic activity of human gut microbes has been suggested to represent a virtual organ within an organ [2,3]. In recent years, this hidden metabolic organ [4]. has received renewed research interest for potential associations with human health. This idea already dates back to the early 1900s, when Metchnikoff first postulated the role of the gut in host physiology and pathology [5]. He proposed that gut bacteria are essential modulators influencing homeostasis and that deregulation of gut homeostasis by certain bacteria leads to a diseased state owing to poisoning of the body from bacterial byproducts.

Indeed, the importance of disruption of the gut microbiota, or so-called dysbiosis, is now recognized in various conditions such as obesity [6,7,8,9,10], type 2 diabetes mellitus (T2DM) [11,12], chronic inflammatory diseases like inflammatory bowel disease [13], and colorectal cancer [14,15]. Moreover, clinical studies indicate that the administration of beneficial microbes via direct supplementation [16]. and/or fecal microbial transplantation [11]. can significantly modulate host hepatic and systemic lipid metabolism [9], energy balance [17], and glycemic control [18], which may reduce diet-induced obesity, insulin resistance, and T2DM. Although the underlying mechanisms require further investigation, there is an increasing body of evidence that the functional output of the gut microbiota, in particular bacterial metabolites like short chain fatty acids (SCFA) and amino acids, are important modulators of host physiology [19]. For example, the SCFA receptor GPR43 has been reported to link the metabolic activity of the gut microbiota with host energy homeostasis [20,21]. GPR43-deficient mice are obese on a normal diet, whereas mice overexpressing GPR43 remain lean even when fed a high-fat diet. Raised in the absence of microorganisms, both types of mice show a normal phenotype; SCFA-mediated activation of GPR43 appears to regulate fat accumulation in adipocytes and energy expenditure in other tissues, including liver and muscle, in order to maintain energy homeostasis [21].

Furthermore, studies in germ free and conventionalized mice reveal that gut bacteria alter the distribution of free amino acids in the gastrointestinal (GI) tract [22]. This may suggest that the gut microbiota affects the bioavailability of amino acids to the host. Interestingly, amino acids can also serve as precursors for the synthesis of SCFA by bacteria [23], suggesting an interplay between microbial activity and host amino acid and SCFA homeostasis. Importantly, emerging evidence links obesity, insulin resistance, and T2DM to elevated systemic concentrations of a small cluster of amino acids, the branched-chain amino acids (BCAA), in human adults [24]. These amino acid alterations are likely to contribute to the development of metabolic disease. For example, infusion of a cocktail of 18 amino acids including the BCAA leucine, isoleucine and valine has been shown to lead to decreased insulin sensitivity in man [25]. In view of the above, it is of interest to discuss microbial amino acid metabolism in the human GI tract in relation to its potential role in the development of obesity and related metabolic disorders. Hence, the objectives of this review are to compile the available evidence on the contribution of microbial amino acids to host amino acid homeostasis, and to assess the potential role of the gut microbiota as a determinant of amino acid and SCFA perturbations in human T2DM and obesity.

## 2. Microbial Amino Acid Metabolism

### 2.1. Amino Acid Fermenting Microbes in the GI Tract

The GI tract is the habitat of a highly diverse and dynamic microbial community, whose metagenome outnumbers the human genome over one hundred times [2]. This complex community is predominantly composed of bacterial species. The activities of these bacteria have potential effects on host nutrition and health through the metabolism of dietary components and through interaction with the intestinal epithelium [26]. As part of the major nutrients in the diet, amino acids should be particularly taken into account since they not only support the growth and survival of bacteria in the GI tract [27], but also regulate energy and protein homeostasis in organisms [28,29]. An early study already contributed to the hypothesis that gut bacteria may play an important role in host amino acid homeostasis and health by showing that germ free mice had an altered distribution of free amino acids along the GI tract as compared to conventionalized mice [30]. Along the GI tract, alimentary and endogenous proteins are hydrolyzed into peptides and amino acids by host- and bacteria-derived proteases and peptidases [22,31]. The released peptides and amino acids can be further utilized by both gut bacteria and the host. The most abundant amino acid fermenting bacteria in the human small intestine are bacteria belonging to the *Clostridium* clusters, the *Bacillus-Lactobacillus-Streptococcus* groups, and *Proteobacteria* [32]. In the large intestine of healthy humans, bacteria belonging to the *Clostridia* and *Peptostreptococci* appear to be the most prevalent species involved in amino acid fermentation [23,32,33]. These bacteria are therefore likely to be important for protein digestion and subsequent amino acid absorption in the GI tract [23]. Interestingly, intrabacterial amino acid composition varies among bacterial strains. Gut bacteria generally contain a higher proportion of BCAA relative to other amino acids [32]. Whether this may indicate increased BCAA synthesis and/or uptake, or decreased BCAA breakdown, and if this influences host BCAA availability remains to be determined.

### 2.2. Microbial Amino Acid Catabolism and Utilization

When comparing amino acid absorption to the capacity of enterocytes for amino acid metabolism, it appears that the gut microbiota is likely to utilize certain amino acids in the small intestinal lumen. Upon uptake by bacteria, amino acids can be either directly incorporated into bacterial cells as protein building blocks, or become catabolized. In an early study in milk-fed piglets [34], it was already proposed that the small-intestinal microbiota uses lysine. Lysine catabolism in the intestinal mucosa was found to be quantitatively greater than lysine incorporation into mucosal proteins. More recently, the same was suggested for other amino acids like methionine and phenylalanine which appear to be partly utilized by the small-intestinal microbiota as well [23]. Indeed, the amounts of methionine in the vena porta were found to only represent approximately half of the dietary methionine in piglets, suggesting that part of the dietary methionine is consumed in the GI tract. In agreement with these results, amino acid catabolism by the small-intestinal microbiota has been confirmed by *in vitro* studies where single amino acids were metabolized by both monocultures and mixed bacterial cultures derived from the pig small intestine [35,36]. Consistent with this, bacteria harbor highly active peptidases [37]. Although protein breakdown followed by amino acid absorption in the small intestine is a rather efficient process, substantial amounts of amino acids seem to escape assimilation in the small intestine in humans [38]. These amino acids can subsequently be used by the microbiota in the colon, or transported from the lumen into the portal blood stream. In addition, the host itself produces substrates such as glycoproteins (e.g., mucins) which contribute to the available amino acids within the colon [39]. Interestingly, the mucin-degrading bacterium Akkermansia muciniphila has been shown to affect insulin sensitivity in mice [40], although it is unclear if this involves mucin amino acid degradation.

In contrast to the proximal part of the intestine where the concentrations of proteins, peptides, and amino acids are relatively high, bacterial concentrations are greater in the more distal part of the intestine of humans. Partly due to these differences in microbiota abundance and composition along the GI tract, bacterial amino acid metabolism in the gut is likely to be compartment specific. Regarding the large intestine, it appears that amino acids are not significantly absorbed by the colonic mucosa, but rather are intensively metabolized by the large intestinal microbiota [23]. This higher rate of bacterial protein fermentation has been related to high pH and low carbohydrate availability in the large intestine [22]. The preferred amino acid substrates of colonic bacteria include lysine, arginine, glycine, and the BCAA leucine, valine, and isoleucine [32], resulting in the generation of a complex mixture of metabolic end products including among others ammonia, SCFA (acetate, propionate, and butyrate), and branched-chain fatty acids (BCFA; valerate, isobutyrate, and isovalerate). Importantly, these bacterial metabolites have been shown to influence epithelial physiology by influencing signaling pathways in epithelial cells and by modulating the mucosal immune system of the host [41,42]. Besides, they also modulate bacterial gene expression leading to the production of enzymes involved in amino acid metabolism [43].

Next to the generation of SCFA and BCFA, microbial metabolism of amino acids can also give rise to biogenic amines. Biogenic amines are produced by decarboxylation of amino acids. The biogenic amines mainly produced by the resident microbiota include cadaverine (a decarboxylation product of lysine) and agmatine (a decarboxylation product of arginine) [44]. These biogenic amines can have significant physiological effects *in vivo*. For example, agmatine was recently shown to influence multiple physiological and metabolic functions in rats by elevating tissue cAMP levels, ultimately replicating the effects of caloric restriction with respect to metabolic reprogramming and leading to reduced diet-induced weight gain [45].

Furthermore, in a study with obese hypertensive individuals, consumption of the probiotic *Lactobacillus plantarum* (which decarboxylates ornithine to produce putrescine) resulted in a reduced body mass index and lower arterial blood pressure [46]. The urinary putrescine levels in these obese individuals were significantly higher and were positively associated with higher *Lactobacilli* counts. Another microbial-derived amine produced by decarboxylation of histidine is histamine. Histamine has potent immunoregulatory effects via the activation of histamine receptors, and there is experimental evidence that a *L. rhamnosus* strain exerts anti-inflammatory effects by activating histamine receptors [47].

Overall, amino acids can be either utilized for the synthesis of bacterial cell components, or catabolized through different pathways. This diversity of amino acid metabolism in gut bacteria may have either positive or negative effects on the host. Consequently, modulating dietary protein or amino acid intake may provide a strategy for shaping the amino acid fermenting bacteria and their metabolic pathways, thereby potentially affecting host metabolism.

### 2.3. Microbial Amino Acid Biosynthesis

Next to utilizing amino acids, bacteria appear to play an important role in the production of amino acids as well by means of *de novo* biosynthesis. For example, *in vitro* studies have shown that ruminal bacteria, such as *Streptococcus bovis*, *Selenomonas ruminantium,* and *Prevotella bryantii* perform *de novo* synthesis of amino acids in the presence of physiological concentrations of peptides [48]. *In vivo* studies have also shown that microbial derived lysine, which is an essential amino acid, is absorbed and incorporated into host proteins [49,50,51]. Comparison of the incorporation of ^15^N from ^15^NH_4_CL into body lysine in germ-free and conventionalized rats indicated that all ^15^N-lysine detected was from microbial origin [52]. In a follow-up study, these researchers determined that approximately 75% of the microbial ^15^N-labeled lysine was absorbed by the small intestine [53]. In a pig model, the synthesis of essential amino acids by gut bacteria and their absorption were assessed from the incorporation of ^15^N from dietary ^15^NH_4_Cl and of ^14^C from dietary ^14^C-polyglucose into amino acids in body tissues [54]. Because pig tissues cannot incorporate ^15^N into lysine or ^14^C into essential amino acids, the detected incorporation in body protein indicated a microbial origin. In accordance with these animal studies, the intake of oral ^15^N in the form of ^15^NH_4_Cl in six healthy men appeared to contribute to the labeling of microbial protein, and threonine from intestinal microbial origin appeared in the portal blood stream *in vivo* [55]. Moreover, a significant contribution of microbial derived lysine and threonine to the free plasma lysine and threonine pool was observed in adult humans on nitrogen adequate diets [50]. Additionally, the same research group recently found a significant contribution of microbial lysine to the body protein pool in humans [28]. In support, it has been determined that amino acids in plasma can derive from microbially degraded urea in human infants [51]. Furthermore, it has been reported that the microbiota in the large intestine is enriched with genes involved in essential amino acid biosynthesis using precursors derived from the plasma pool in humans [53]. In fact, biosynthetic genes for the essential amino acid threonine have recently been identified in the human gut microbiome [56]. However, the net microbial contribution to whole body amino acid metabolism in humans remains uncertain because interpretation of results obtained by the ^15^N approach is complicated due to nitrogen recycling into and from the gut [28].

### 2.4. Amino Acids as Precursor for Microbial-Derived SCFA

SCFA are traditionally considered the end products of bacterial fermentation of dietary fibers and resistant starch in humans and other mammals [57,58]. The most abundant SCFA are acetate, propionate and butyrate. In addition, formate, valerate, caproate, 2-methyl-butyrate, and isovalerate are produced by gut bacteria, but in considerably smaller quantities. Metabolic intermediates formed during colonic fermentation can be ultimately metabolized to SCFA as well due to metabolic interactions among gut bacteria (*i.e.*, cross-feeding) [59]. Once absorbed, butyrate is mainly used as energy source by the colonic epithelium. The remaining butyrate is used by liver cells for gluconeogenesis and cholesterol synthesis, along with propionate. Acetate, on the other hand, is mainly utilized by muscle cells to generate energy [58]. Interestingly, it has been reported that undigested proteins and amino acids in the colon may serve as an additional substrate for SCFA production next to nondigestible carbohydrates [60,61,62]. Indeed, various amino acids produced from microbial protein fermentation in the large intestine can serve as precursors for SCFA synthesis [63]. Amino acids utilized by anaerobic bacteria that can be metabolized to acetate include glycine, threonine, glutamate, lysine, ornithine, and aspartate [63]; threonine, glutamate, and lysine can be used for the synthesis of butyrate. Propionate has been reported to be mainly produced from threonine [23]. Thus, among the amino acids used for SCFA synthesis, threonine is the most versatile, giving rise to all three major SCFA. BCAA, on the other hand, do not appear to function as precursors for SCFA synthesis. Furthermore, the addition of BCAA (valine, leucine, and isoleucine) to trypticase yeast extract has been shown to increase the yield of BCFA in *Clostridia* [64]. These observations provide evidence that several amino acids can be used for SCFA production as well as for BCFA production by gut bacteria.

## 3. Gut Microbiota and Its Relation with Obesity

Changes to lifestyle and an increase in the availability of energy-rich foods are important contributors to the worldwide obesity epidemic. The gut microbiota should probably be considered an additional environmental factor contributing to the development of obesity and its comorbidities. Indeed, as said before, gut bacteria are increasingly recognized to modulate metabolic processes. The gut microbial communities are dominated by five bacterial phyla (*Firmicutes*, *Bacteroidetes*, *Actinobacteria*, *Proteobacteria* and *Verrucomicrobia*) of which up to 90% of the species belong to the *Firmicutes* and *Bacteroidetes* [65]. Whereas each individual harbors a distinct and highly diverse microbiota [66], the core microbiota and genes are shared among individuals [2,7].

However, in obese humans, gut microbiota composition is significantly different from that in normal weight individuals [6]. The fecal microbiota in obese humans shows a shift towards more *Firmicutes* and fewer *Bacteroidetes* [7,67]. Similar changes in the abundance of these dominant gut phyla, with a 50% reduction in the abundance of *Bacteroidetes* and a proportional increase in *Firmicutes*, have been observed in obese (*ob*/*ob*) mice [68]. Interestingly, in obese humans, levels of *Bacteroidetes* have been shown to increase upon diet-induced weight loss [7,69,70,71]. The mouse gut microbiota is similarly responsive to diet-induced weight loss, leading to a larger proportion of *Bacteroidetes* and relatively fewer *Firmicutes* [72]. In addition, corresponding increased levels of *Bacteroidetes* have been observed in patients who lost weight by gastric bypass surgery [73]. Collectively, these findings indicate that some types of bacteria correlate with body weight. However, it should be noted that there have also been contradictory studies that did not find a shift in the *Firmicutes-Bacteroidetes* ratio [74,75]. Others even reported increases in species belonging to both *Firmicutes* and *Bacteroidetes* in overweight women [76]. These discrepancies may be related to the use of different criteria for determining obesity and, particularly, different microbiota-profiling methodologies.

Nevertheless, the role of the gut microbiota in determining energy harvest from food and subsequent fat deposition has been clearly demonstrated using germ-free rodents [8,9]. First, germ-free mice showed reduced adiposity compared to conventionalized mice. Upon conventionalization, adiposity was normalized in these mice without any increase in food consumption, indicating that the gut microbiota directly affect energy harvest and fat storage. Moreover, fecal excretory energy losses of germ-free rats have been shown to be much greater compared to conventionalized rats fed the same diet [77]. In order to compensate for the reduced energy harvest, germ-free rats showed an almost 20% increased intake of calories. This implies that there is a specific microbiota that obtains more energy from the same caloric intake. Similar observations were reported in ob/ob mice compared to their lean counterparts in microbiota transplantation experiments. Bomb calorimetry revealed that ob/ob mice have significantly less energy remaining in their faeces [8]. Importantly, the microbiota of these obese mice was rich in genes encoding enzymes that break down nondigestible dietary carbohydrates leading to more fermentation end-products (SCFA) and less energy remaining in the faeces. Another interesting finding from the microbiota transplantation experiments in mice is that obesity can be transmitted by transplantation of microbiota, supporting that altered gut microbiota composition is a cause instead of a consequence of obesity [8]. In line, from a study in children, it was concluded that changes in gut microbiota composition precede weight changes [78]. Moreover, transplantation of “lean microbiota” into the gut of obese humans has been shown to be associated with metabolic improvements including reduced insulin resistance [11].

Subsequent studies proposed several other mechanisms by which gut microbiota composition changes could contribute to the development of obesity [17,79]. One of these mechanisms concerns modulation of energy expenditure through fatty acid oxidation and energy storage in the form of triglycerides. Gut bacteria have been shown to alter fatty acid metabolism promoting fat accumulation in the liver and adipose tissue of mice. In detail, intestinal secretion of an inhibitor of adipose lipoprotein lipase (LPL) called fasting-induced adipose factor (FIAF) was suppressed in mice upon conventionalization, increasing the storage of triglycerides [17]. Other studies suggested a role for gut microbiota in modulating nutrient uptake via the signaling action of SCFA. SCFA are ligands for two G-protein-coupled receptors (GPCRs) Gpr41 and Gpr43, expressed by gut epithelial and enteroendocrine cells, but also by adipocytes [79,80]. Upon binding, these receptors are activated and induce the secretion of the gut hormones glucagon-like peptide-1 (GLP-1) and peptide YY (PYY). PYY regulates intestinal motility which may affect nutrient absorption from the gut, whilst GLP-1 regulates satiety [81]. Hence, both conventionalized Gpr41-deficient mice and germ-free Gpr41-deficient mice colonized with commensals of the human distal gut were shown to be leaner compared to wild type mice [79]. As intestinal transit time is inhibited by PYY, Gpr41-deficient mice showed decreased energy harvest from the diet. Thus, the gut microbiota may, via this mechanism of SCFA-mediated GPCR activation, contribute to increased nutrient uptake and deposition [82].

Recent studies have also shown that overweight and obese humans had higher fecal SCFA concentrations than their lean counterparts on a similar diet, confirming that colonic fermentation also differs according to body weight in humans [75,83]. Higher fecal SCFA concentrations could result from decreased SCFA absorption and/or from increased SCFA production. As mentioned before, the latter could be related to increased conversion of amino acids into SCFA. In other words, the obesity-associated changes in SCFA levels may reflect increased microbial amino acid catabolism. In line with this hypothesis, modulation of the gut microbiota by antibiotics has been shown to increase plasma amino acid concentrations in piglets compared with controls [84]. Furthermore, there is a marked increase in the portal concentrations of several essential amino acids during high-fat diet induced obesity and glucose intolerance [85]. It could also be speculated that chronic elevations in systemic BCAA levels, as seen in obesity [86], impair transport of these amino acids from the intestinal lumen into the systemic circulation, thereby contributing to persistent increased amino acid catabolism in the lumen and more SCFA formation.

Taken together, the gut microbiota is increasingly being accepted as an environmental factor that affects nutrient acquisition, energy harvest, and many host metabolic pathways. Given the evidence discussed above, the potential impact of gut microbiota activity on both amino acid and SCFA perturbations in obesity warrants further investigation.

## 4. Gut Microbiota and Its Relation with Amino Acid Perturbations in Type 2 Diabetes Mellitus

Concomitant with the rising prevalence of obesity, T2DM rates show a dramatic increase, affecting over 6% of the world population. Like obesity, T2DM has recently been found to be characterized by an altered intestinal microbiota composition [2,87,88]. Recent work particularly emphasizes the relevance of the innate immune system in the pathogenesis of T2DM [89,90]. Animal studies have demonstrated that modulation of gut microbiota composition upon high-fat feeding is associated with a significant increase in inflammatory status [91]. In obese subjects with impaired glucose tolerance, similar associations were demonstrated as obesity specific intestinal microbiota composition seemed to be related to both intestinal and systemic inflammation [92]. Moreover, transplantation of fecal microbiota from lean individuals into obese subjects resulted in improved insulin sensitivity [11]. Also, gastric bypass surgery has been reported to have direct antidiabetic effects probably due to a shift in the composition of the gut microbiota [10,73,93]. Thus, it is likely that gut microbiota alterations play an important role in the pathogenesis of human obesity-induced T2DM as well. However, as with the study of obesity, the mechanisms underlying the effects of the gut microbiota on the development of human T2DM require further clarification.

As described above, plasma levels of certain amino acids are elevated in obese, insulin resistant, and type 2 diabetic individuals [94,95,96]. In particular, increases in a small cluster of essential amino acids including the BCAAs (*i.e.*, leucine, valine, and isoleucine), and the aromatic amino acids (*i.e.*, phenylalanine and tyrosine), have been shown to be associated with a ~5-fold increased risk of developing T2DM in the future [24]. Furthermore, plasma BCAA levels were shown to be predictive of T2DM even after adjustment for age, sex, BMI, physical activity, alcohol intake, smoking, systolic blood pressure and HDL cholesterol, suggesting that BCAAs may exert independent effects on insulin resistance and T2DM risk [97]. These amino acid alterations are likely to actively contribute to the development of metabolic disease, since supplementation of BCAAs to a high-fat diet has been shown to lead to the development of insulin resistance in rats [25].. Moreover, infusion of a cocktail of 18 amino acids in humans has also been shown to decrease insulin sensitivity, further supporting that amino acids contribute to the development of metabolic diseases. On the other hand, feeding mice a leucine-depleted diet has been shown to result in improved insulin sensitivity [98]. In support, the well-known improvement of glycemic control in obese subjects after gastric bypass surgery is paralleled by a massive decline in circulating BCAA (phenylalanine and tyrosine), which is much greater than the decline observed in subjects that lost an equal amount of weight through diet intervention [99]. Taken together, there is accumulating evidence that next to gut microbiota alterations, changes in plasma levels of BCAAs and aromatic amino acids are associated with insulin resistance and T2DM.

Though the causes of the amino acid derangements within T2DM still have to be elucidated, gut microbiota have been shown to be important factors for the supply of both aromatic amino acids and BCAAs including leucine, phenylalanine, isoleucine, and valine to mammalian hosts [28]. In addition, intestinal bacteria are known to have a major impact on nutrient absorption [6,71,100]. Therefore, it is conceivable that the altered bacterial composition in the gut of subjects with T2DM contributes to their amino acid derangements. Additional evidence for this view comes from a recent large case-cohort study that demonstrated only a modest association between dietary protein intake and risk of developing T2DM [101], consistent with the concept that dietary protein does not significantly contribute to the amino acid changes seen in this population.

Proteolytic activity in the large intestine leading to the generation of amino acids has been mainly attributed to the genera *Bacteroides*, *Clostridium*, *Propionibacterium*, *Fusobacterium*, *Streptococcus*, and *Lactobacillus* [22]. Of these, *Clostridium* bacteria species have been reported to be especially prevalent in T2DM [12]. Furthermore, feeding a high-fat diet to mice harboring *Clostridium* ramosum in the gut gave rise to a remarkable 2 to 4-fold higher rate of weight increase compared to mice fed a high-fat diet without this *Clostridium* member in their intestines [102]. This is consistent with *Clostridium* mediated proteolysis leading to increased amino acid and possibly SCFA levels in T2DM.

The potential importance of SCFAs in T2DM has been underscored by the clinical observation that alpha-glycosidase inhibitors, which are frequently prescribed as antidiabetics, influence plasma SCFA concentration and total fecal SCFA output [103,104]. Reducing the abundance of gut microbiota by antibiotics treatment has also been shown to result in fluctuations in the levels of fecal SCFA as well as fecal amino acids [105]. This suggests that antibiotics induce a change in bacterial protein fermentation and/or protein degradation. In this regard, a strong correlation between fecal amino acids and fecal SCFA profiles and the abundance of certain bacterial groups has been reported. While the levels of amino acids, including the BCAA leucine, isoleucine and valine, the aromatic amino acids (tyrosine), and other amino acids (alanine, lysine, and methionine), and SCFA (acetate, propionate, and butyrate) were positively correlated with *Prevotella*, *Alistipes* and *Barnesiella*, they were negatively associated with *Bacteroides* and *Enterococcus*. Interestingly, *Prevotellaceae* have been reported to be significantly enriched in obesity [10]. Moreover, the abundance of *Alistipes* has been shown to decrease by modulation of the gut microbiota in parallel with alleviation of T2DM [106]. This could be related to effects of *Alistipes* on amino acid levels. Next to *Alistipes*, *Barnesiella* has been suggested to have pro-diabetogenic effects as well [107].

## 5. Conclusions

The intestinal microbiota is involved in the utilization and catabolism of several amino acids originating from both alimentary and endogenous proteins. These amino acids can serve as precursors for the synthesis of metabolic end products produced by the microbiota including SCFAs. T2DM has been characterized by systemic elevations in some of these (precursor) amino acids. The altered bacterial composition in the gut as observed in obese subjects with T2DM may therefore play a major role in their metabolic derangements by influencing amino acid and SCFA bioavailability to the host.
